# In Vitro Evaluation of Cytotoxicity and Permeation Study on Lysine- and Arginine-Based Lipopeptides with Proven Antimicrobial Activity

**DOI:** 10.3390/molecules22122173

**Published:** 2017-12-08

**Authors:** Malgorzata Anna Dawgul, Katarzyna Ewa Greber, Sylwia Bartoszewska, Wioletta Baranska-Rybak, Wieslaw Sawicki, Wojciech Kamysz

**Affiliations:** 1 Department of Inorganic Chemistry, Faculty of Pharmacy, Medical University of Gdansk, Hallera 107, 80-416 Gdansk, Poland; sylwiabart@gumed.edu.pl (S.B.); kamysz@gumed.edu.pl (W.K.); 2 Department of Physical Chemistry, Faculty of Pharmacy, Medical University of Gdansk, 80-416 Gdansk, Poland; greber@gumed.edu.pl (K.E.G.); wsawicki@gumed.edu.pl (W.S.); 3 Department of Dermatology, Faculty of Medicine, Medical University of Gdansk, 80-210 Gdansk, Poland; wiolabar@gumed.edu.pl

**Keywords:** lipopeptides, antimicrobial peptides, toxicity, keratinocytes, skin permeation

## Abstract

Owing to their excellent antimicrobial activities with a relatively low cost of production, lipopeptides are being intensively investigated as potential alternatives to popular antimicrobials. However, a critical obstacle for their application is a relatively high toxicity, hence a lot of attention has been paid to designing new molecules with optimal properties. In this study we synthesized the following lipopeptides: C_16_-KK-NH_2_, C_16_-KεK-NH_2_, C_16_-KKK-NH_2_, C_16_-KRK-NH_2_, C_16_-RR-NH_2_, C_16_-RRR-NH_2_, (C_10_)_2_-KKKK-NH_2_ and (C_12_)_2_-KKKK-NH_2_. Their antimicrobial activity against representative strains of Gram-positive bacteria, Gram-negative bacteria and fungi has been confirmed. The compounds have been evaluated with regard to the safety of their application in dermatology. The cytotoxicity was determined in HaCaT keratinocytes using MTT assay, whereas Strat M membranes placed in Franz diffusion cells were used to assess their ability to skin permeation. The compounds containing one hexadecanoic acid chain turned out to be very toxic towards human keratinocytes, while lipopeptides containing two fatty acid chains (decanoic and dodecanoic) demonstrated much lower cytotoxicity. For the most promising lipopeptide, (C_10_)_2_-KKKK-NH_2_, the measured IC_50_ on HaCaT keratinocytes was few times higher as compared to MICs obtained for the tested bacteria. Both groups of lipopeptides did not permeate the model membranes and therefore lack of permeation through human skin could be expected. The results of this work encourage further research on the potential application of lipopeptides with two fatty acids as novel antimicrobials.

## 1. Introduction

Antimicrobial peptides (AMPs) are considered a promising alternative to conventional antimicrobials. There are numerous studies confirming their effectiveness against a broad spectrum of drug resistant microbes, activity against microbial biofilms and low rate of microbial resistance development. Despite numerous advantages of AMPs, there are also many limitations for their therapeutic applications. The most significant ones are a relatively low bioavailability and high costs of production [1,2]. A lot of attention has been paid to designing new analogues: shorter compounds containing the sequence crucial for antimicrobial action or designing novel compounds basing on native AMPs as well as new high throughput methods for their assessment [3,4,5]. To obtain compounds with improved stability, several approaches have been applied: advanced drug formulation or modification of chemical structure of compounds, such as introduction of D-amino acid or fatty acid residues into the molecule [6,7].

Short lipopeptides are amphipathic, positively charged molecules exhibiting antimicrobial activity of AMPs being less cost- and time-consuming in production. They are composed of short peptide chains containing positively charged amino acids (arginine, lysine, and ornithine) combined with a fatty acid residue (palmitic, myristic, lauric) [8,9].

According to the literature, short lipopeptides exhibit strong antimicrobial activity against a broad spectrum of microorganisms including numerous bacteria and fungi [10].

In this study we synthesized six short lipopeptides composed of short peptide chains (containing lysine and/or arginine residues) combined with hexadecanoic (palmitic) fatty acid and two lipopeptides containing a peptide chain composed of four lysine residues combined with two residues of decanoic (capric) and dodecanoic (lauric) acids. The compounds were subjected to an in vitro evaluation with regard to their potential application in dermatology.

## 2. Results

### 2.1. Antimicrobial Activity

All tested compounds exhibited relatively strong antibacterial and moderate antifungal activities (Table 1). *S. aureus* was inhibited after application of peptides at a concentration of 2 to 16 mg/L. The most active compound in the MIC assay on *S. aureus* was C_16_-RRR-NH_2_, while the lipopeptide (C_12_)_2_-KKKK-NH_2_ exhibited the weakest activity (MIC = 16 mg/L). For *E. coli*, somewhat higher concentrations of lipopeptides were required (MIC for the majority of compounds extended from 8 to 16 mg/L). As in the case of Gram-positive bacteria, the lowest activity was exhibited by a lipopeptide containing two residues of the lauric acid (MIC = 64 mg/L). The growth of the representative of fungi, *C. albicans*, was inhibited by the peptides at significantly higher concentrations. The MICs for the majority of lipopeptides was 128 mg/L, while for the lauric acid derivative it was 256 mg/L. The MBC/MFC values were mostly twice as those of MICs, indicating their bactericidal and fungicidal activities.

### 2.2. Cytotoxicity towards HaCaT Cells and Permeation Study

The tested compounds exhibited different cytotoxicity in HaCaT cells depending on their chemical structure.

Derivatives of palmitic acid were very cytotoxic and the measured IC_50_ value ranged from 1.8 to 7.4 mg/L (Table 2), which in the case of all lipopeptides was below the lowest microbiologically active concentration. The most toxic peptides were C_16_-KK-NH_2_ and C_16_-RR-NH_2_. In this group of compounds, the lowest toxicity displayed a lipopeptide with two lysine residues where the peptide bond was created with the ε-amine group of the second residue of lysine.

In contrast to lipopeptides with the hexadecanoic acid, the compounds containing two chains of decanoic and dodecanoic acids turned out to be non-toxic towards human keratinocytes at their microbiologically active concentrations. The measured IC_50_ values for (C_10_)_2_-KKKK-NH_2_ and (C_12_)_2_-KKKK-NH_2_ were 49.4 mg/L and 42.1 mg/L, respectively. For the first peptide, the toxic concentration is as high as about a few times the MICs of compound obtained using bacterial strains.

During the permeation experiments, none of the tested compounds was found in the acceptor solution. On the basis of the signal to noise ratio, the limit of detection of the compounds was calculated. The minimum detectable concentration was 1 mg/L. Therefore, we can expect that the compounds would not be able to penetrate the *stratum corneum* at concentrations at which they are harmful to the living keratinocytes.

## 3. Materials and Methods

### 3.1. Lipopeptide Synthesis

Lipopeptides were obtained via solid phase synthesis on solid polymer support. For protection of α-amino groups, 9-fluorenylmethoxycarbonyl (Fmoc) was applied. The protocol was described in the previous paper [11]. The compounds were purified with reversed phase high-performance liquid chromatography. Their purity and identity were confirmed by RP-HPLC and MALDI-TOF mass spectrometry, respectively. The compounds were obtained in order to assess their toxicity and perform the skin permeation study. Their synthesis was previously described when the compounds were obtained for microbiological studies [12,13,14]. The sequences and molecular masses of the compounds are presented in the Table 3.

### 3.2. Antimicrobial Activity

The compounds were dissolved in a mixture of dimethyl sulphoxide (DMSO) and phosphate buffer (PBS). The final concentration of DMSO in the inoculated samples did not exceed 10%. It has been confirmed that this concentration of DMSO does not influence the growth of tested microorganisms. Application of 10% DMSO allowed to obtained solutions of C_16_-KK-NH_2_, C_16_-KKK-NH_2_ and C_16_-KRK-NH_2_ at concentration of 512 mg/L, arginine based lipopeptides precipitated at the highest concentration, while compounds with two residues of fatty acids precipitated at 512 mg/L and 256 mg/L. For obtained solutions/suspensions the minimum inhibitory concentration (MIC) and minimum bactericidal (MBC) and fungicidal concentrations were determined (MFC). MIC was determined by the serial dilution method on polystyrene 96-well plates with Mueller–Hinton II broth (MHB II) for reference bacterial strains: *Staphylococcus aureus* (ATCC 25923) and *Escherichia coli* (ATCC 25922), while for *Candida albicans* (ATCC 10231), the *Sabouraud* dextrose broth was applied. The microorganisms were obtained from the Polish Collection of Microorganisms (Wroclaw, Poland). Bacteria were cultured at 37 °C under aerobic conditions overnight in Brain Heart Infusion broth, centrifuged at 1000 rpm for 8 min, washed thrice with PBS and resuspended in MHB II. *Candida albicans* was cultured in *Sabouraud* dextrose broth for 48 h at 27 °C under aerobic conditions, centrifuged at 1000 rpm for 8 min, washed thrice with PBS and resuspended in *Sabouraud* dextrose broth. Bacterial (5 × 10^5^ CFU/mL) and fungal (5 × 10^4^ CFU/mL) suspensions were exposed to lipopeptides applied at graded concentrations (range 1–512 mg/L) for 18 h at 37 °C under aerobic conditions (bacteria) and 48 h at 27 °C under aerobic conditions (fungi). MIC was taken as the lowest concentration at which no visible growth of bacteria/fungi was observed. MBC and MFC were determined using MH II agar and *Sabouraud* Dextrose agar, for bacteria and fungi respectively. The contents of wells with MICs and two subsequent higher concentrations were transferred to agar plates and cultivated for 18 h at 37 °C under aerobic conditions (bacteria) and 48 h at 27 °C under aerobic conditions (fungi). The MBC and MFC were taken as the lowest concentrations at which no colony forming units were found. All tests were performed in triplicate.

### 3.3. Cell Line, Culture Conditions and Cytotoxicity Assay

HaCaT cells were obtained from American Type Culture Collection (ATCC). Cells were cultivated in Dulbecco’s modified Eagle Medium (Invitrogen) with 10% fetal bovine serum in humidified incubator at 39 °C in 5% CO_2_ and cultured in 96-well plates and allowed to grow to 60–70% confluence prior to the start of the experiments.

The MTT assay is a colorimetric technique for assessing cell metabolic activity based on NAD(P)H-dependent cellular oxidoreductase enzymes activity that, under defined conditions, reflects the number of viable cells present. Viable cells reduce the tetrazolium dye MTT 3-(4,5-dimethylthiazol-2-yl)-2,5-diphenyltetrazolium bromide to its insoluble formazan, which has a purple colour. The colour intensity reflects the number of live cells and can be measured spectrophotometrically. Briefly, one day after culturing 500 cells per well, different concentrations of compounds were applied. After a 24-h incubation with the specified compounds, a medium containing 1 mg/mL MTT was added to the cells (HaCaT keratinocytes) up to a final concentration of 0.5 mg/mL and incubated at 37 °C for 4 h. The medium was then aspired, and the formazan product was solubilized with DMSO. The absorbance at 630 nm (background absorbance) was subtracted and absorbance measured at 570 nm for each well. There were 6 replicates for each tested concentration. All experiments were repeated at least twice. The resulting IC_50_ values were calculated with a GraFit 7 software (v. 7.0, Erithacus, Berkley, CA, USA).

### 3.4. Permeation Study

The study was performed on Strat M membranes (Merck, Milipore, Burlington, MA, USA) placed in Franz diffusion cells (SES, Bechenheim, Germany). The compounds at concentrations of 1000 mg/L in ethanol-water solution (20% *m*/*m*) were added on the surface of the membranes (1.12 ± 0.03 cm^2^) and incubated for 24 h. The acceptor solution was the phosphate buffer of pH 7.4 (V = 3.79 ± 0.03 mL). The samples (200 µL of acceptor solution) were taken after 0.5, 1, 2, 4, 8, 12 and 24 h of incubation at 32 ± 1 °C. The samples were analyzed by RP-HPLC with a linear gradient 2-98% of solvent B in solvent A in 20 min, eluents: (A) 0.1% trifluoroacetic acid (TFA) in water, (B) 0.1% TFA in acetonitrile (ACN), flow rate: 2.0 mL/min, UV detection: 214 nm, column: Chromolith Performance C18e 4.6 mm × 100 mm. A calibration curve was based on peak area measurements of lipopeptide solutions with concantrations ranging 0.01 ÷ 10.000 mg/mL with R^2^ ≥ 0.9991. The limit of detection (LOD) for tested peptides was found to be around 1.00 μg/mL; the limit of reasonable quantification (LOQ) was set at 3.00 μg/mL.

## 4. Discussion

Since the introduction of lipopeptides as a potential alternative to available antimicrobials, they have become a topic of intensive research around the globe. Excellent antimicrobial activities with relatively low production costs have drawn the attention of many research groups to investigate lipopeptides as a potential alternative for antibiotic therapy. Numerous studies focusing on their antimicrobial activities and designing molecules with optimal properties have been conducted. 

C_16_-KK-NH_2_ belongs to a group of the extensively studied compounds. In the so far performed studies, the peptide demonstrated high antimicrobial activity against Gram-positive cocci, including antibiotic-resistant strains [13]. Our previous research on palmitic acid derivatives revealed their high effectiveness against biofilm formed by the clinical strains of *Staphylococcus aureus*, comparable to that of vancomycin [14]. Moreover, the C_16_-KK-NH_2_ peptide enhanced the activity of the antibiotic used in prophylaxis of vascular graft infection in the rat model [15]. Serrano et al. synthesized short-sequence peptides containing alanine, glycine, leucine and lysine combined with palmitic acid and demonstrated their strong activity against the strains of *S. aureus*, both MSSA as well as MRSA [16]. Similar activity was presented by lipopeptides composed of tryptophan and ornitine residues combined with capric, caproic, caprylic, lauric, myristic and palmitic acids [17]. Combination of lauric acid with short sequences composed of ornithine and cysteine resulted in a high anti-staphylococcal activity against clinical strains [14]. Application of another lipopeptide based on lauric acid, C_12_-CKK-NH_2_, in combination with daptomycin reduced the development of resistant strains of *Enterococcus faecalis* [18]. In our previous study, peptides C_16_-KK-NH_2_ and C_16_-RR-NH_2_ did not induce the development of resistance by *S. aureus* strains isolated from patients with atopic dermatitis [19]. Those findings support the hypothesis about the low risk of microbial resistance development and membrane related mechanism of action proposed for lipopeptides [9]. In our study, the lipopeptides demonstrated relatively high antimicrobial activities and moderate anti-fungal activities. 

The results of the MTT assay obtained in this study indicate high toxicity of lipopeptides containing one palmitic acid chain. This contradicts the previously advanced hypotheses about the selectivity of antimicrobial peptides. According to some authors, peptides designed based on naturally occurring compounds in human organisms are expected to be safe to human cells and their selectivity results from the presence of cholesterol and arrangement of lipids in the eukaryotic cell membrane [20,21]. Our results suggest non-selective membrane activity of palmitic acid derivatives. In the previous study, peptide C_16_-KK-NH_2_ also exhibited a negative influence on proliferation and viability of human keratinocytes at very low concentrations (from 1 mg/L) [22]. There is no data on cytotoxicity of remaining palmitic acid based lipopeptides towards HaCaT cells, but their toxicity towards eukaryotic cells had been confirmed previously by hemolytic assays. The lipopeptides disrupt the membranes of red blood cells once they are exposed to the compounds at concentrations close to their MICs [9,11]. Those findings and our results are not encouraging for continued research on this group of lipopeptides and their potential application in the therapy of infections. However, the results of the permeation study allow to expect that the compounds could be applied on intact skin without the risk of harm to living deeper layers of skin or systemic adverse effects. The used synthetic membranes are composed of two layers of polyethersulfone which are more resistant to diffusion and one layer of polyolefin characterized by an enhanced permeability. The polymeric layers create a porous structure with an increasing size of pores and permeability mimicking human skin. The results obtained previously by the producer confirm a high correlation of the results obtained with Strat M membranes and human skin in comparison to animal skin models [23]. Therefore, the lack of penetration through human skin after topical application of the peptides can be expected. Based on the obtained results and the data from previous studies, we suggest that lipopeptides containing arginine and lysine residues combined with palmitic acid could be further tested for their potential application as topical treatment for *S. aureus* carriage. Moreover, it is worth mentioning that a successful in vivo study on C_16_-KK-NH_2_ has been performed. Its effectiveness in the prevention of staphylococcal biofilm infection in a rat model has been confirmed [14]. Therefore, it can be hypothesized that the cytotoxity of the peptides is reduced in physiological environments. It has been found that the toxicity of the peptides is inhibited by the plasma ingredients such as apolipoproteins A–I and B [20,21]. Due to the discrepancies between in vivo and in vitro toxicities, the obtained results do not exclude palmitic acid derivatives from further studies. However, it seems reasonable to focus the attention on the lipopeptides containing two fatty acid chains. For instance, lipopeptide (C_10_)_2_-KKKK-NH_2_ not only presents a high antimicrobial activity against a broad spectrum of bacteria, but also is non-toxic towards HaCaT keratinocytes and human red blood cells at its microbiologically active concentrations [12]. According to the classification of antimicrobial peptides presented by Shai and Abrahami in patent on antimicrobial and anticancer peptides, the compound can be defined as highly active against *S. aureus* and active against *E. coli* [24]. The results of this work encourage continued research on the potential application of lipopeptides as novel antimicrobials. Our further research will focus on this group of compounds as tools for prevention and elimination of bacterial biofilm as well as on designing new analogues with optimal biological properties: high antimicrobial activity and low toxicity towards human cells.

## Figures and Tables

**Table 1:** Minimum inhibitory concentrations (MIC) (mg/L) and minimum bactericidal/fungicidal concentrations (MBC/MFC) (mg/L) of the lipopeptides.

	*S. aureus*		*E. coli*		*C. albicans*		Reference
Compound	MIC	MBC	MIC	MBC	MIC	MFC	
C_16_-KK-NH_2_	4	4	8	8	128	128	[11]
C_16_-KεK-NH_2_	8	8	16	32	128	256	-
C_16_-KKK-NH_2_	4	8	8	16	128	128	[11]
C_16_-KRK-NH_2_	4	16	8	16	128	256	-
C_16_-RR-NH_2_	8	8	16	16	128	256	-
C_16_-RRR-NH_2_	2	4	16	16	128	256	-
(C_10_)_2_-KKKK-NH_2_	8	16	16	16	128	256	[12]
(C_12_)_2_-KKKK-NH_2_	16	32	64	64	256	512	[12]

**Table 2:** Values of IC_50_ (mg/L) and their standard deviations obtained for the tested compounds on HaCaT keratinocytes exposed to their action for 24 h.

Compound	IC_50_ ± SD
C_16_-KK-NH_2_	1.8 ± 0.2
C_16_-KεK-NH_2_	7.4 ± 0.9
C_16_-KKK-NH_2_	3.2 ± 0.1
C_16_-KRK-NH_2_	2.3 ± 0.4
C_16_-RR-NH_2_	1.9 ± 0,3
C_16_-RRR-NH_2_	3.2 ± 1.4
(C_10_)_2_-KKKK-NH_2_	49.4 ± 9.1
(C_12_)_2_-KKKK-NH_2_	42.1 ± 9.1

**Table 3:** Amino acid sequences and molecular masses (Da) of the lipopeptides.

Amino Acid Sequence of Lipopeptide		Molecular Mass
Lipopeptide 1	C_16_-KK-NH_2_	511.6
Lipopeptide 2	C_16_-KεK-NH_2_	511.6
Lipopeptide 3	C_16_-KKK-NH_2_	639.9
Lipopeptide 4	C_16_-KRK-NH_2_	667.9
Lipopeptide 5	C_16_-RR-NH_2_	567.8
Lipopeptide 6	C_16_-RRR-NH_2_	723.7
Lipopeptide 7	(C_10_)_2_-KKKK-NH_2_	837.5
Lipopeptide 8	(C_12_)_2_-KKKK-NH_2_	893.6
